# Directing and Potentiating Stem Cell-Mediated Angiogenesis and Tissue Repair by Cell Surface E-Selectin Coating

**DOI:** 10.1371/journal.pone.0154053

**Published:** 2016-04-22

**Authors:** Zhao-Jun Liu, Pirouz Daftarian, Letícia Kovalski, Bo Wang, Runxia Tian, Diego M. Castilla, Emre Dikici, Victor L. Perez, Sapna Deo, Sylvia Daunert, Omaida C. Velazquez

**Affiliations:** 1 Department of Surgery, School of Medicine, University of Miami, Coral Gables, Florida, 33136, United States of America; 2 Department of Biochemistry and Molecular Biology, School of Medicine, University of Miami, Coral Gables, Florida, 33136, United States of America; 3 Dr. JT Macdonald Biomedical Nanotechnology Institute, University of Miami, Coral Gables, Florida, 33136, United States of America; 4 Department of Ophthalmology, Bascom Palmer Eye Institute, University of Miami, Coral Gables, Florida, 33136, United States of America; Universidad de Castilla-La Mancha, SPAIN

## Abstract

Stem cell therapy has emerged as a promising approach for treatment of a number of diseases, including delayed and non-healing wounds. However, targeted systemic delivery of therapeutic cells to the dysfunctional tissues remains one formidable challenge. Herein, we present a targeted nanocarrier-mediated cell delivery method by coating the surface of the cell to be delivered with dendrimer nanocarriers modified with adhesion molecules. Infused nanocarrier-coated cells reach to destination via recognition and association with the counterpart adhesion molecules highly or selectively expressed on the activated endothelium in diseased tissues. Once anchored on the activated endothelium, nanocarriers-coated transporting cells undergo transendothelial migration, extravasation and homing to the targeted tissues to execute their therapeutic role. We now demonstrate feasibility, efficacy and safety of our targeted nanocarrier for delivery of bone marrow cells (BMC) to cutaneous wound tissues and grafted corneas and its advantages over conventional BMC transplantation in mouse models for wound healing and neovascularization. This versatile platform is suited for targeted systemic delivery of virtually any type of therapeutic cell.

## Introduction

Success of stem cell therapy relies on efficient engraftment of viable cells to a diseased tissue, through either local or systemic route, to achieve the desired therapeutic effect and restore tissue homeostasis and function. Currently, the most widely used route of stem cell administration is direct injection of cells into the diseased tissue. However such an application poses significant limitations. In general, retention and survival of injected cells are poor [1]. The major causes of poor survival of stem cells *in vivo* are linked to anoikis, potential immune rejection, and oxidative damage mediating apoptosis [2]. In addition, injected cells may not survive or function due to an unfavorable local microenvironment, such as tissue physical pressure caused by limited space within a given tissue where exogenous cells are forcibly inoculated, or lack of sufficient nutrition and oxygen. Furthermore, many intra-cavitary injured or disease areas, such as brain, chest, abdomen and pelvis, may not be safely accessed via invasive inoculation techniques. In contrast, systemic delivery of therapeutic cells, which is accomplished through the circulatory system using physiological mechanisms whereby endogenous circulating stem cells home to injured areas, does not have these limitations, thus potentially results in a more widely applicable approach. However, the number of cells that home to the targeted tissues following this approach is, in general, significantly less than that transplanted by local injection [3]. Hence, it is critical to develop methods for specific systemic delivery that yield a sufficient number of viable cells to targeted diseased tissues.

Luminal endothelial cells (EC) form the natural barrier between the blood and surrounding tissue. In steady-state physiological conditions, EC are mostly quiescent and form an impermeable or lowly-permeable barrier dependent upon tissues. Under pathological conditions, such as tissue injury, inflammation and tumors, a variety of cytokines/chemokines, for example, SDF-1α, TGF-ß, and IL-1, are released into tissue, and the local endothelium is stimulated by these soluble factors. This results in upregulation and/or activation of a unique panel of cell adhesion molecules (CAMs), including selectins and integrins, in the endothelium within the local tissue. This causes EC to switch from an impermeable/lowly-permeable to highly-permeable and ‘sticky’ status. These adhesion molecules act as “docking” sites and facilitate tethering of circulating inflammatory, immune-modulatory and repair cells, such as bone marrow-derived endothelial progenitor cells (EPC) and mesenchymal stem cells (MSC). The “docked” circulating cells undergo tight adhesion to the endothelium and subsequent transendothelial migration, extravasation from highly-permeable capillaries/postcapillary venules, and infiltration into dysfunctional tissues [4]. We and others demonstrated that up-regulated E-selectin on luminal EC in cutaneous wound tissues or tumor tissue is responsible for mediating EPC homing [5–7]. E-selectin is an inducible cell-adhesion molecule expressed on endothelium and binds to P-selectin glycoprotein-1 (PSGL-1/CD162), CD44, and E-selectin ligand (ESL-1), presented on the surface of various circulating cells [8]. E-selectin is also presented in certain types of circulating cells, for example EPC, and responsible for EPC homing via binding to its counterpart ligands expressed on activated capillary endothelium [5]. The presence of CD162 on the endothelium [9], in particular, on the endothelial lining of atherosclerotic coronary arteries [10] has been observed, suggesting a role in the formation of the inflammatory infiltrate in these types of diseased or inflamed arterial wall lesions. Indeed, endothelial CD162 plays a crucial role in mediating rolling and adhesion of platelets and peripheral blood mononuclear cells over activated endothelium [10]. Thus, one can envision these existing physiologic and/or pathologic mechanisms being exploited for delivery of repair cells, i.e. certain vascular adhesion molecule pairs (receptor/ligand), which are expressed on circulating cells and/or luminal EC could be utilized to direct therapeutic stem cells homing to sites of injury or disease. However, “installing” desired adhesion molecule(s) on the cell surface via a biological approach, such as gene expression or mRNA-based transient expression, can raise safety concerns due to side-effects associated with induced non-directional differentiation and others resulting from viral-vectors employed in gene transfer [11]. Lipid insertion method [12] is a potential option, yet the quick internalization rate and uneven cell surface distribution may limit its application. An alternative strategy for targeted cell delivery includes the creation/expression of an engineered non-natural membrane molecule, e.g. vesicular stomatitis virus glycoprotein that recognizes the specific corresponding molecule in tumor tissue under low pH [13]. However, potential applications of such an approach are diminished by limitations imposed by the need for low pH in the desired tissue repair site; such an approach would not be feasible in repair of myocardium, cornea, cutaneous wounds, and injured arteries, among others. Other known targeted cell-delivery methods [14,15] rely on the innate capability of immune cells to recognize antigens expressed on targeted cells/tissues, thus limiting applicability of these methods to immune therapies.

Herein we describe an alternative strategy that employs modified nanocarrier with programmed molecular recognition moieties for targeted cell delivery. Specifically, we coated the surface of stem cells with polyamido amine (PAMAM) dendrimer nanocarriers that are complexed with adhesion moieties/molecules. These modified nanocarriers interact with the cell membrane through ionic interactions (dendrimer are highly-positive charged while the cell membrane is negatively charged), thereby installing the adhesion molecules on the cell surface. The nanocarriers employed in our work were specifically modified to achieve optimal cell surface coating with minimal intracellular internalization and no cell toxicity. The adhesion molecule moiety was selected and designed based upon availability of counterpart adhesion receptor or ligand highly or selectively expressed/activated on the endothelium within targeted diseased tissue (in this work, surgically created cutaneous and corneal wounds). This approach thus augments capture of the coated cells at the microcirculatory bed of the desired site. The captured cells can then undergo extravasation and influx into the targeted tissue intended for a healing, angiogenesis, and regeneration. The graphic abstract is an Illustration of our proposed directed cell therapy platform and its biological effects. We showed biosafety of the nanocarriers *in vitro* and *in vivo* and demonstrated efficient and specific delivery of coated stem cells to cutaneous wound tissues and injured cornea selectively without being ‘captured’ in common cell-sink organs such as lung, liver, and spleen. Thereafter we observed these cells eliciting a marked therapeutic biological response at the injured sites in mouse models. Thus, our targeted nanocarrier technology provides a highly specific and biocompatible approach that significantly enhances targeted tissue homing for potentially broadly applicable regenerative medicine applications.

## Materials and Methods

### Acetylation of G5 dendrimers (Ac-G5) and conjugation of protein(s) with Ac-G5

The ratio of acetic anhydrides and the dendrimer (CAS Number 163442-68-0, Sigma-Aldrich, St Louis, MO) was adjusted to achieve ~30% of amine groups to be acetylated. To 15 mL of anhydrous methanol, 1 mL of 5 wt% dendrimer in methanol and triethylamine (6.8 μL, 48.71 μmol, 28.15 eq) were added and stirred for 30-min. Then, acetic anhydride (4.2 μL, 44.29 μmol, 25.6 eq) was added to the reaction mixture, overnight at room temperature (RT) under an argon atmosphere. Evaporator removed the methanol, and the residue was dissolved in water. Dialyis against 1 L of PBS for 8-hour and then against H_2_O (3 x 8 h) in a 10-KDa cutoff Slide-A-Lyzer Dialysis cassette (Pierce Biotechnology, Rockford, IL). The samples were then lyophilized and stored at 4°C. Proton nuclear magnetic resonance spectra were taken in D_2_O using a Varian 400 MHz spectrophotometer, using the solvent as reference. The extent of primary amine acetylation was determined from the NMR data.

sE-sel and VEGF (10335-H08H and 10008-HNAB, Sino Biological Inc., Beijing, China) were reconstituted in H_2_O (0.66 mg/mL and 0.4 mg/mL, respectively). Ac-G5, sE-Sel and VEGF were separately diluted to different concentrations in Opti-MEM Reduced-Serum Medium (Invitrogen, Grand Island, NY) to give different mole ratios of dendrimer:protein ranging from 10:1 to 1:10. The protein was added to Ac-G5 and incubated for 15-min at RT for complexation.

### Cell culture and coating cells with nanocarriers

HUVEC (PCS-100-010; ATCC, Manassas, VA) and human EPC (NDRI, Philadelphia, PA) were cultured as described [6]. MSC (which do not express E-selectin as measured by immunoblot, data not shown) were obtained by culture of bone marrow-derived mononuclear cells (BMC) from ROSA26 (LacZ^+^) mice (B6.129S7-GT (ROSA)26sor/J, Jackson Lab, Stock# 002192) with MesenCult® medium (#05501 & #05502; StemCell Technologies, Vancouver, Canada). MSC cultured for 2-weeks with periodic medium changes (every 2 days) were characterized as CD73^+^/CD105^+^/Lin^-^ (data not shown). MSC (LacZ^+^) were then transduced with luciferase2 (Luc2)/Lentivirus for cell tracking. All cells were incubated at 37°C in 98% humidified air containing 5% CO_2_. To coat cells, 5 x 10^6^ cells were mixed with dendrimer-protein complex suspended in 1 mL PBS for 30-min at RT with a periodical shaking (every 5-min). Cells were washed with 3 mL PBS (X3) before use.

### Transmission electron microscopy (TEM) and confocal imaging to detect cell surface-bound nanocarriers

For the TEM experiments, MSC coated with nanocarrier/sE-sel or control complexes were collected and washed with precooling PBS twice and then fixed in 2.5% glutaraldehyde at 4°C for 2 h. The cells were post-fixed in a slide in 1% osmium tetroxide at 4°C for 1 h and then washed with PBS for 20 min twice. The cells were then stained with 0.5% aqueous uranyl acetate at room temperature overnight. To dehydrate the slides, they were immersed in 30%, 50%, 70%, and 90% ethanol for 10 min at each percent and then 100% ethanol for 10 min twice. Samples were embedded in Epon812 and cut into ultrathin sections. Sections were collected onto grids and stained with 1% (w/v) uranyl acetate and lead citrate. Samples were observed under a JEM-1200EX electron microscope (Nihon Kohden Corporation, Tokyo, Japan). For the confocal microscopy, MSC were seeded as sub-confluent on 24-well glass bottom plates (P24G-1.5-10-F, MatTek, Ashland, MA) pre-coated with Poly-L-Lysine for overnight. Dendrimer-protein complexes of different mole ratios were added to the cells and allowed to incubate for 90-min at 37°C. Cells were then washed with PBS twice, and imaged with a Leica SP5 confocal microscope. Z-stacks were 25 μm thick with each slice picture taken at 0.5 μm intervals.

### Recombinant lentiviruses and cell transduction

DsRed/lenti and Luc2/lenti were constructed by inserting cDNA into *pLenti6* (Invitrogene, Grand Island, NY). Production of pseudotyped lentivirus was achieved by co-transfecting 293T cells with three plasmids [16,17]. To infect cells, cells were exposed for 6-hour to lentivirus with multiplicity of infection 5 with 4 μg/mL polybrene (Sigma-Aldrich). Cells were cultured for two additional days, and analyzed by fluorescence microscope (DsRed/lenti transduced cells) or bioluminescence detector (Luc2/lenti transduced cells). Cells were pooled for subsequent analysis as indicated in individual experiments.

### *In vitro* EPC-EC interaction assay

1 x 10^5^ cells/well of HUVEC were cultured in the 24-well glass plates pre-coated with 1% gelatin and cells reached 100% confluence one-day later. The HUVEC monolayers were stimulated with recombinant human SDF-1α (R & D Systems, Minneapolis, MN) or BSA at 100 ng/mL for 8-hour. Nanocarriers-coated DsRed^+^ EPC (1 x 10^5^) were suspended in 1 mL basal EGM2 medium. To test the effect of E-selectin antagonist on EPC adhesion to SDF-1α-stimulated EC, E-selectin neutralizing antibody (BBA1; R & D Systems) or isotype-matched control antibody (2 μg/mL) were added into EC monolayer and incubated for 15-min before adding nanocarriers-coated EPC. HUVEC-EPC were co-cultured for 1-hour at 37°C. Unbounded EPC were washed out twice with 1 mL PBS. Fluorescence signals derived from adherent DsRed^+^ EPC were measured and quantified by fluorescence scanner (GE Typhoon Trio, Piscataway, NJ).

### *In vivo* local and systemic cell injection and β-Galactosidase assay and IHC

All animal studies were done with approval from the University of Miami Institutional Animal Care and Use Committee. Creation of cutaneous wounds, measurement of healing rate, β-galactosidase assay and IHC were described previously [6,18]. For local wound tissue cell injection, 1×10^6^ MSC from 8~12-week old *db/db* mice (non-diabetes yet) were coated with or without either Ac-G5-sE-sel or Ac-G5-BSA, and injected directly into >18-week old diabetic *db/db* mice that had dorsal cutaneous full-thickness 6-mm punch biopsy wounds. For systemic cell injections, 1×10^6^ MSC (suspended in 100 μL saline) from 10~12-week old Rosa26(*LacZ*^+^) mice were coated with or without either Ac-G5-sE-sel or Ac-G5-BSA and injected via tail vein into C57BL/6 mice that had dorsal cutaneous full-thickness 6-mm punch biopsy wounds. Cell injection was conducted 2-hour after creation of the skin wounds. The number of recruited MSC was quantified by counting LacZ^+^ cells in serial sections of excisional wounds at POD 8 in 5 random high power fields (HPF, 40X) per section in at least 3 serial sections. To stain blood vessels, tissue sections were incubated with HRP-anti-CD31 (ab28364; Abcam, Cambridge, MA) or isotype matched control antibody for overnight at 4°C (n = 6/group). To detect CD44 expression in capillaries of wound tissues, tissue sections were co-stained with FITC-CD44 and Alexa Fluor^®^594-KDR using immunostaining method described previously [6,18]. All mice employed in this study were maintained at the DVR animal facility under standard conditions. Mice were anesthetized for all surgical procedures by ketamine/xylazine mixture (100/10 mg/kg, IP), and imaging procedures by inhaling 3% isoflurane gas, and sacrificed in CO2 chamber. Institutional animal care and use committee at the University of Miami approved all animal procedures (#14–127).

### Bioluminescence imaging for monitoring biodistribution of infused MSC in wounded mice

Mice were anesthetized with isoflurane; D-luciferin was injected intraperitoneally (i.p.) 10-min prior to imaging (150mg/kg). Animals were imaged using the IVIS 200B with a 1–3 min capture, medium binning. Regions of interest were drawn and quantified using the Living Image software. Bioluminescence signal was reported as total light emission within the region of interest (photon/s). A signal was defined as positive when it was greater than the sum of the mean + 2 standard deviations (SD) of the background signal.

### Orthotopic corneal transplantation and BMC injections

Mice were anesthetized (i.p.) with Ketamine (100 mg/kg) and Xylazine (10 mg/kg) before the surgical procedure. Orthotopic syngeneic corneal transplantations were performed with C57BL/6 as recipients and donors [19]. Corneal grafts with flat anterior chamber, ulceration or other complications related to surgical difficulties were excluded. All corneal sutures were removed at POD 7. On POD 14, 1×10^6^ BMC (suspended in 100 μL saline) from EGFP^+^ transgenic mice (Gt(ROSA)26S or-EGFP) [20] coated with either Ac-G5-BSA or Ac-G5-sE-sel were injected (i.v.) (n = 5/group). The next day, the corneas of live mice under (2% isoflurane) anesthesia were imaged.

### DiI perfusion, confocal miscroscopy and *in vivo* imaging of the corneal grafts

Corneal grafts were imaged longitudinally as previously described [21]. For imaging analysis, Z-stack images acquired using the resonant scanner were denoised and contrast-enhanced using Volocity software (PerkinElmer, Waltham, MA). The z-stack thickness was adjusted to span the whole thickness of the imaged cornea. Corneal and diabetic wound tissue blood vessels were directly labeled by live perfusion using a specially formulated aqueous solution (7 mL/mouse) containing DiI (D-282, Invitrogen) as previously reported [18,22]. Seven mL of 4% paraformaldehyde was injected following DiI perfusion and the entire eye was harvested. The vascular network was visualized by scanning the entire eye or wound to a depth of 200 μm using scanning confocal microscopy. Vessel density was quantified when Leica SP5 confocal 3D images were imported and volume of positive signal was quantified using Improvisions Volocity 6.3.

### Statistical analyses

Statistical analysis of differences was performed using ANOVA and 2-tailed Student’s *t*-test for paired comparison. Data are expressed as mean ± SD. Values are considered statistically significant when *p*<0.05.

## Results

### Determination of optimal cell surface coating conditions

**Fig 1:**
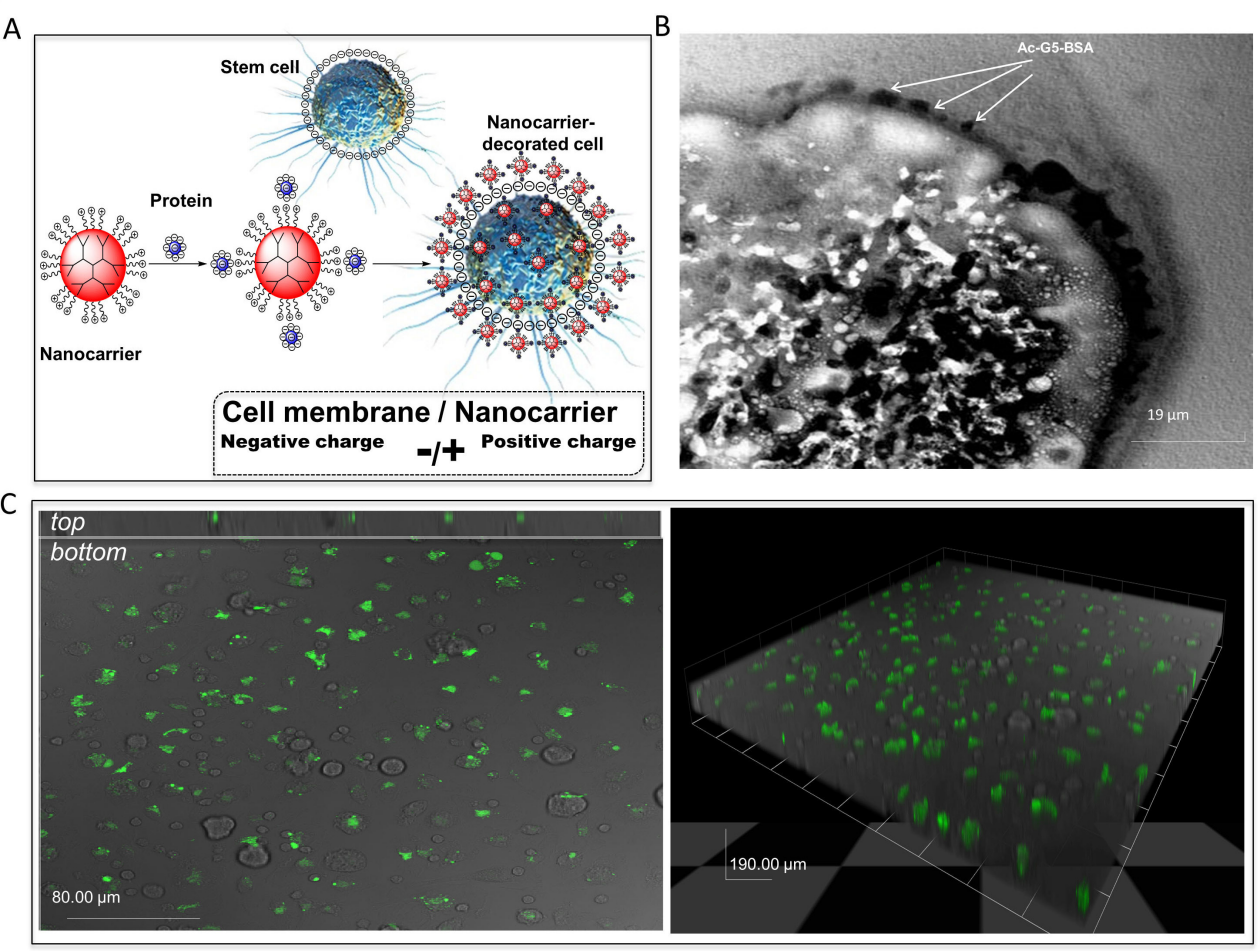
Coating cell surface with nanocarriers. **A**. Schematic illustration for the design of nanocarrier for cell coating. **B** & **C**. Murine MSC were coated with albumin-Alexa-488 conjugated Ac-G5-dendrimer nanocarriers and subjected to image analysis 3-hour afterward. Cell membrane binding and the sustained surface stay of the nanocarriers were assessed by 2 methods. TEM imaging is shown in **B**. Arrows point to Ac-G5-dendrimer-BSA binding on the cell surface. Confocal imaging is shown in **C**. *Left*: Top: a orthogonal view of stacks of confocal images showing Ac-G5-dendrimer-BSA localization at the cell surface; Bottom: a surface confocal imaging of coated MSC (X40). *Right*: 3D overview of confocal z-stack imaging of coated MSC (X40).

PAMAM dendrimers have emerged as attractive nano-vehicles with low toxicity and biocompatibility [23,24]. Dendrimers are highly branched macromolecules spanning from a central core and containing a series of layers that are structurally and synthetically distinct. Each layer added to the structure of a dendrimer is referred to as a “generation” (G). Thus far, dendrimers have been primarily used for targeted delivery of various therapeutic agents, including drug, DNA, or protein [25,26]. Typical nanocarrier targeted delivery systems consist of dendrimer vehicles that carry the therapeutic agent and recognition elements that direct the vehicle to the desired location. Upon recognition, the nanocarriers anchor the therapeutic agent to the cell membrane of the target tissue, but to date there are no reports on transport of therapeutic cells for cell-based therapy. Herein, we investigated the characteristics G5-dendrimer as nanocarriers for targeted delivery of therapeutic cells. Specifically, the negatively charged stem cell surface was coated with the positively charged nanocarriers through ionic interactions (Fig 1A). To prolong cell surface coating duration and avoid internalization of the nanocarrier into the coated cells, we determined optimal condition which decreases the overall positive charge of the G5-dendrimer, while retains cell surface binding capability, by acetylating ~30% of the positively charged amines. The Ac-G5 nanocarriers were used to coat mouse bone marrow-derived MSC (BM-MSC) as described in the Methods. Optimization of the complexation protocol prolonged cell surface coating up to 3-hour, which was confirmed by TEM (Fig 1B) and confocal fluorescence experiments (Fig 1C). Z-stacks showed that Ac-G5-albumin-FITC nanocarriers stayed on the surface of BM-MSC for 3-hour with minimal internalization, making it ideal for our studies. To minimize cellular toxicity, we determined optimal dendrimer modification conditions and ratio of dendrimer or Ac-dendrimer to protein to be conjugated. We then examined the cytotoxicity of various dendrimers and modification conditions toward human umbilical vein endothelial cells (HUVEC). Cell viability assays demonstrated that Ac-G5 nanocarriers with 30% acetylation did not cause HUVEC cytotoxicity (Table A in S1 File), which is consistent with previous work showing that acetylation of dendrimers reduces cell toxicity [27]. Collectively, these experiments identified Ac-G5 as the optimal nanocarrier for our studies given the long cell surface retention time, lack of cytotoxicity, and minimal internalization.

### Verification of efficient cell-cell adhesion with nanocarriers *in vitro*

**Fig 2:**
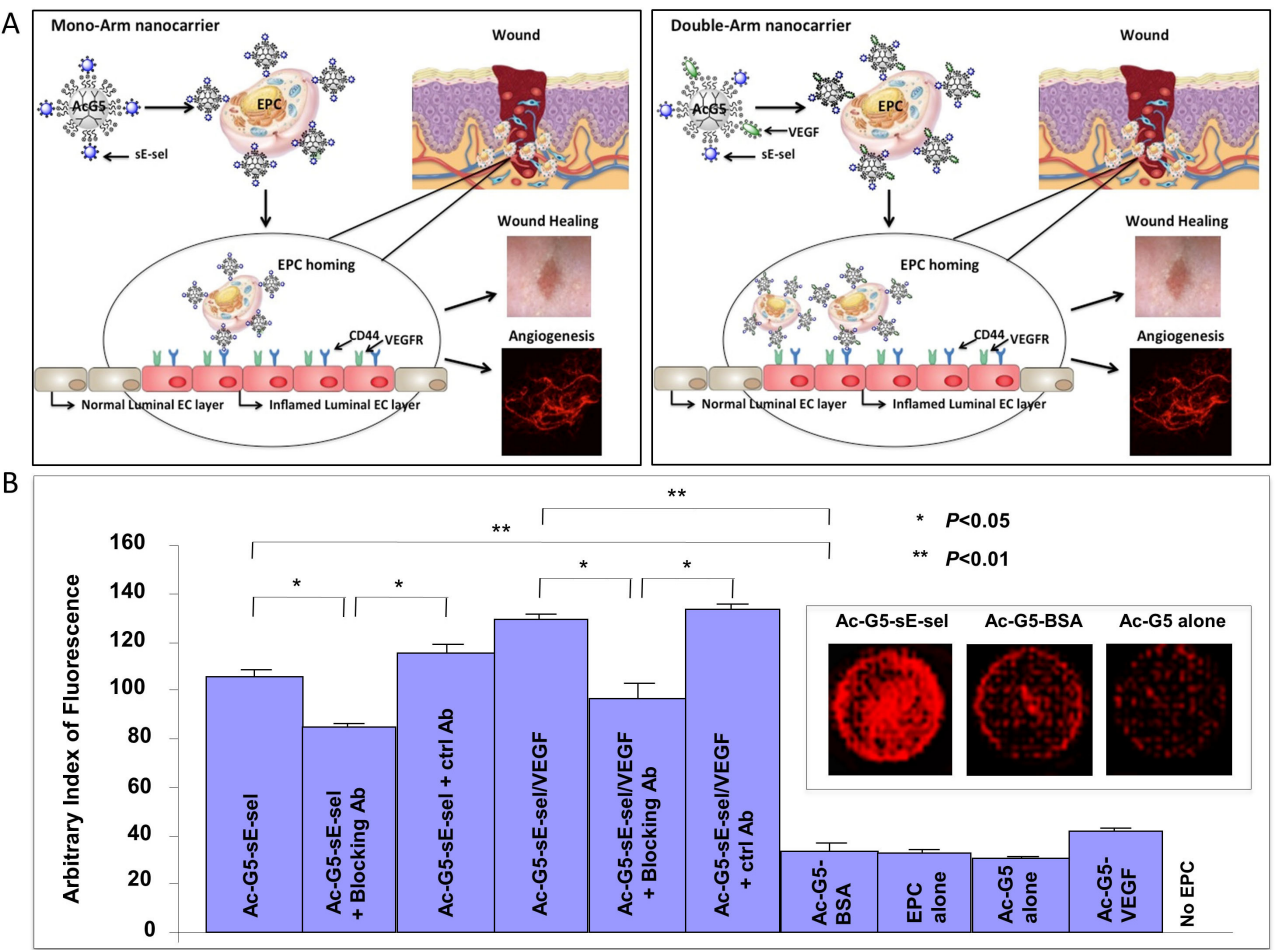
Nanocarrier-mediated cell-cell binding *in vitro*. A. Schematic illustration of the design of nanocarriers with a representative image of the desired biological readout. “Mono-arm” (Ac-G5-dendrimer-sE-sel, *Left*) and “double-arm” (Ac-G5-dendrimer-sE-sel/VEGF, *Right*) for EPC coating. **B.** *In vitro* EPC-EC binding assay. “Mono-arm” and “double-arm” nanocarrier-decorated DsRad^+^ EPC displayed an increased binding capability to EC monolayer. E-selectin blocking antibody inhibited nanocarrier-mediated association of EPC-EC. Quantitative data of DsRed signals. Data are presented as mean ± SD of three independent assays in which samples were duplicated. *Insert*: Representative images of DsRed fluorescence in wells.

Next, we prepared a nanocarrier consisting of Ac-G5-dendrimer and adhesion molecules sE-sel and sE-sel/VEGF, and performed *in vitro* cell-cell adhesion experiments to determine its effectiveness in enhancing cell-cell interactions. sE-sel has a theoretical pI value of 5.7. Therefore, it is mostly negatively charged at the physiological pH. This high negative charge density can be exploited for the complexation with positively charged dendrimers. Therefore, when sE-sel was mixed with Ac-G5-dendrimer they will form a complex held together due to the electrostatic forces. We specifically studied the interaction between human EPC and human mature EC (EPC-EC adhesion), an essential process involved in the recruitment of circulating EPC to injured tissues. Human EPC, pre-transduced with DsRed/lentivirus (DsRed^+^ EPC) and coated with Ac-G5-sE-sel nanocarrier (“mono-arm construct”) or Ac-G5-sE-sel/VEGF nanocarrier (“double-arm construct”) (Fig 2A), or Ac-G5-BSA nanocarrier (control) were prepared. Subconfluent HUVEC monolayers were stimulated with recombinant human SDF-1α to induce expression of E-selectin ligands, namely CD162 and CD44 [6]. DsRed^+^ EPC coated with different nanocarriers were added to stimulated HUVEC-monolayers; excess unbound DsRed^+^ EPC were washed from the HUVEC monolayers, and presence of adherent DsRed^+^ EPC was imaged by fluorescence (Fig 2B). DsRed^+^ EPC coated with Ac-G5-sE-sel or Ac-G5-sE-sel/VEGF nanocarrier showed superior adhesion to HUVEC monolayers. Application of E-selectin blocking antibody significantly inhibited binding of EPC coated with both “mono-arm” and “double-arm” sE-sel nanocarriers compared to control antibody. Incomplete inhibition is likely due to the efficiency of E-selectin blocking antibody or suggesting that EPC-EC interaction is not fully mediated by E-sel/ligand association. These *in vitro* experiments indicated enhanced sE-sel-decorated nanocarriers mediated EPC-EC interaction.

### Identification of elevated E-selectin ligands on the endothelium of wound bed capillaries

**Fig 3:**
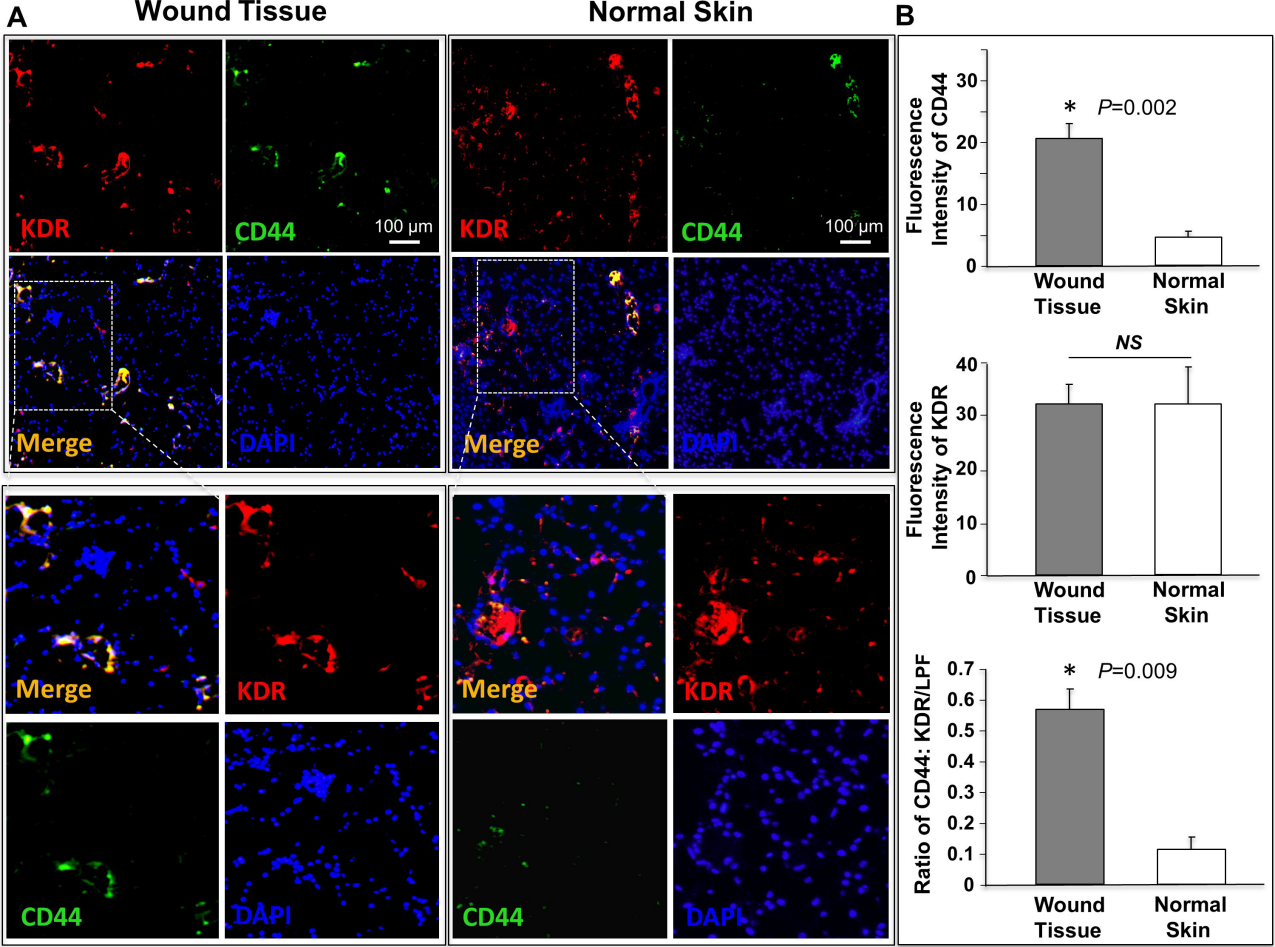
Increased expression of E-selectin ligand, CD44, in luminal EC of skin wounds compared to normal skin. A. Co-expression (yellow) of CD44 (green) and KDR (red) in vessels was detected by immunostaining. Representative images were showed (n = 6 mice/group). The lower panels are enlarged highlighted areas in the upper panels. B. Quantification of CD44 expression in vessels. Data are presented as mean ± SD of KDR (red fluorescence intensity), CD44 (green fluorescence intensity) and ratio of CD44: KDR signals from 5 random selected sections of low power field (LPF, X 10).

The endothelium lining capillary beds of wounded tissues express certain adhesion molecules due to stimulation of inflammatory cytokines/ chemokines, which aids in binding of circulating cells, such as EPC, MSC and inflammatory cells, to the EC lining the wound’s capillaries. To determine the ability of our Ac-G5-sE-sel nanocarriers to direct cells to wounds, we specifically examined expression of the E-selectin ligand CD44 in the microvasculature of surgically induced cutaneous punch biopsies in C57BL/6 mice, since the presence of CD162 has already been demonstrated on injured or inflamed endothelium [10]. We observed that CD44 expression in luminal EC in these cutaneous wounds was significantly higher than in healthy intact skin of the same mice (Fig 3), suggesting that Ac-G5-sE-sel nanocarrier coated cells could improve specific homing to injured skin. Since human and mouse E-selectin share high sequence homology in the lectin-like domain and EGF-like domain, which mediate ligand binding [28], human E-selectin can bind to mouse E-selectin ligands, albeit with a lower affinity [29–32]. Thus, the mouse cutaneous wound model could serve to test the effectiveness of Ac-G5-sE-sel nanocarrier in directing coated cells to target tissue and evaluate pro-angiogenic and pro-repair biological responses.

### Demonstration of wound tissue specificity, pro-angiogenic and pro-repair effects for nanocarrier-mediated delivery of MSC and BMC

**Fig 4:**
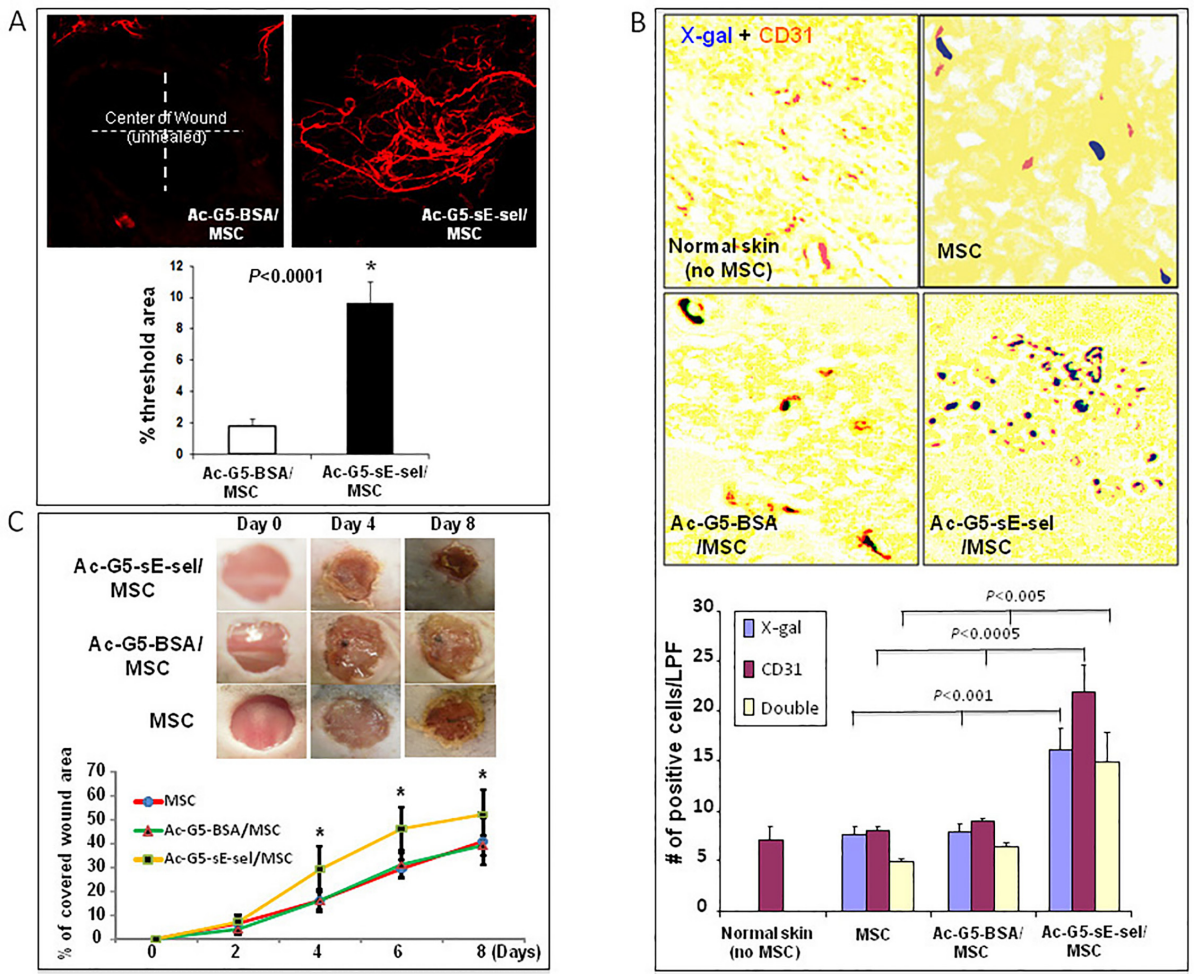
Local and systemic delivery of Ac-G5-sE-sel nanocarrier coated MSC to murine wound tissues. **A**: Pro-angiogenic effect of locally injected Ac-G5-sE-sel nanocarrier coated MSC to wounded db/db mice. 1 x 10^6^ of MSC from donor db/db were coated with Ac-G5-sE-sel or Ac-G5-BSA nanocarriers, and directly injected into wound bed of wounded db/db mice. (*top*) Representative images of confocal microscopy of Dil perfusion; (*bottom*) quantitative data of Dil-stained blood vessel density in wound (n = 6 mice/group). **B**: Homing of systemically delivered Ac-G5-sE-sel nanocarrier coated MSC to wound tissues. 1 x 10^6^ of MSC (LacZ^+^/Luc2^+^) from donor mice were coated with Ac-G5-sE-sel or Ac-G5-BSA nanocarriers, and systemically infused into wounded C57BL/6 mice. (*top*) Representative images of β-gal and CD31 staining (brown) of wound and normal skin tissues. IHC was performed on harvested wounds at day 8 post cell administration in frozen samples that were also subjected to β-gal (blue) staining. Normal skin tissues were used as negative control. (*bottom*) Quantitative data of β-gal^+^, CD31^+^ (single positive) and β-gal^+^/CD31^+^ (double positive) cells (X10). Numbers of positive cells were counted from 5 randomly selected fields in wound and skin samples. Data are percentage of mean ± SD (n = 6 mice/group). **C**. Pro-healing effect of systemic delivery of Ac-G5-sE-sel coated MSC. Healing rate expressed as percent of wound re-epithelialization (covered with new skin). *Top*: representative wounds at different days are shown for each group. *Bottom*: wound healing rate. Data are percentage of mean ± SD (n = 6 mice/group).

We first explored local delivery of the MSC coated with Ac-G5-sE-sel nanocarrier to cutaneous excisional wounds created in diabetes mouse (*db/db*) model. 1 x 10^6^ allogeneic MSC were coated with Ac-G5-sE-sel or Ac-G5-BSA (control) nanocarriers and directly injected into wound bed. Pro-repair efficacy was assessed by daily wound area measurements via digital photography. Fourteen days post-wounding, mice were subjected to whole body DiI perfusion. Wound tissues were harvested for laser scanning confocal microscopy to visualize vessel density. In addition to a faster wound-healing rate (Fig A in S1 File), more than 5-fold increased neovascularization was induced in the wound that received MSC coated with the Ac-G5-sE-sel (Fig 4A). These results demonstrated that cell surface decoration with Ac-G5-sE-sel nanocarrier potentiates pro-angiogenic and pro-healing effects of MSC.

Next, we tested the effectiveness of Ac-G5-sE-sel nanocarriers to direct systemically infused MSC into cutaneous wound tissues and execute pro-angiogenic and pro-repair functions. We compared nanocarrier-decorated MSC with conventional MSC therapy. MSC were selected because they are multipotent stem cells that differentiate into a varied cell types to promote tissue regeneration and wound healing, and have shown to be safe and efficacious in early clinical trials [33,34]. MSC (LacZ^+^/Luc2^+^) were coated with Ac-G5-sE-sel or Ac-G5-BSA (control) nanocarriers. Uncoated MSC were also tested since to date only this form of cell delivery has been used in clinical trials. The nanocarrier-coated and uncoated MSC were injected (i.v.) into mice carrying dorsal cutaneous excisional wounds. Pro-repair efficacy for the uncoated MSC versus coated MSC was assessed by daily wound area measurements via digital photography. Eight days post-wounding, mice were sacrificed and wound tissues were harvested and subjected to immunochemistry. Wound healing was confirmed via histology. MSC homing to wound tissues was detected by X-*gal* staining and bioluminescence monitoring. Blood vessels in wound tissues were stained with anti-CD31 antibody. Mice injected with Ac-G5-sE-sel nanocarriers-coated MSC showed significantly increased numbers of LacZ^+^ MSC within their wound tissues. Some of the LacZ^+^ MSC were located in non-vascular sites while many were incorporated into the wound’s blood vessels (Fig 4B), suggesting that MSC may differentiate into the EC or become pericytes. Importantly, mice treated with MSC coated by Ac-G5-sE-sel nanocarrier had a faster healing rate (Fig 4C). In addition, these treated mice displayed increased angiogenesis (CD31^+^ staining in Fig 4B) within the healing wounds, a distinct biological readout that is important to skin wound healing and other regenerative medicine applications.

**Fig 5:**
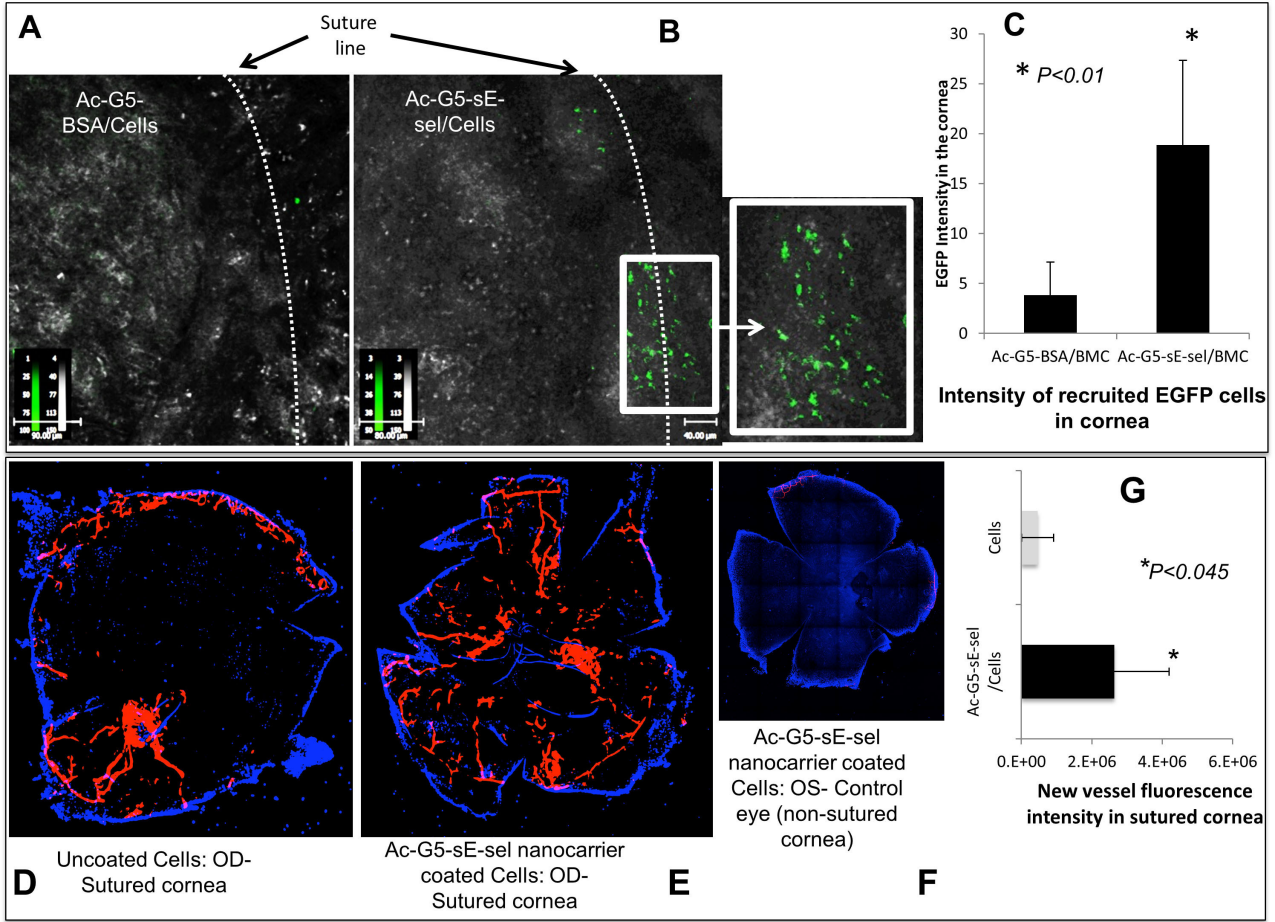
Increased recruitment of systemically infused Ac-G5-sE-sel nanocarrier-coated BMC into grafted corneas enhances neovascularization. *Top*: increased recruitment of Ac-G5-sE-sel coated EGFP^+^ BMC to the grafted cornea compared to cells coated with control nanocarrier. **A & B.** Representative images. The enlarged box is an area along the surgical incision that shows the enhanced recruited cells coated with Ac-G5-sE-sel. **C.** Quantitative intensity of the recruited EGFP^+^ BMC (mean ± SD). *Bottom*: Enhanced corneal neovascularization. Blood vessel density was measured by DiI perfusion and confocal microscopy. **D, E, F.** Representative images of neovascularization 2-days post injection of BMC either uncoated or coated with Ac-G5-sE-sel nanocarrier, respectively (OD (oculus dexter (the right eye))-sutured cornea; OS (oculus sinister (the left eye))-non-sutured eye). **G.** Fluorescent intensity of new-vessels in two groups mean ± SD, n = 5 mice/group.

To explore the versatility of our nanocarrier in specific cell delivery to tissues undergoing damage and repair, we employed a syngeneic corneal transplantation model where the donor and recipient are from the same mice strain known to induce neovascularization [19]. We chose to utilize BMC to test capability of nanocarrier in directing different types of cells. BMC from EGFP transgenic mice were left uncoated or coated with either Ac-G5-sE-sel or Ac-G5-BSA and infused (i.v.) into corneal graft recipients on day 14 of post-corneal transplantation. After 16-hour, *in vivo* confocal images clearly showed a sharp increase in EGFP^+^ BMC in surgically injured corneas of mice that received BMC coated with Ac-G5-sE-sel compared with control nanocarrier (Fig 5A–5C). These cells clustered along the line of surgically created corneal injury. Moreover, on day 2 post-cell-infusion, robustly increased neovascularization was induced in the injured corneas of mice that received BMC coated with the Ac-G5-sE-sel (Fig 5D–5G). These results demonstrated that decorating cells with Ac-G5-sE-sel nanocarrier are superior to traditional methods of cell delivery in mediating targeted cell homing to injured tissue and facilitating tissue-site neovascularization *in vivo* with systemic cell administration.

### Biodistribution of intravenously infused Ac-G5-sE-sel nanocarriers-coated stem cells in wounded mice

**Fig 6:**
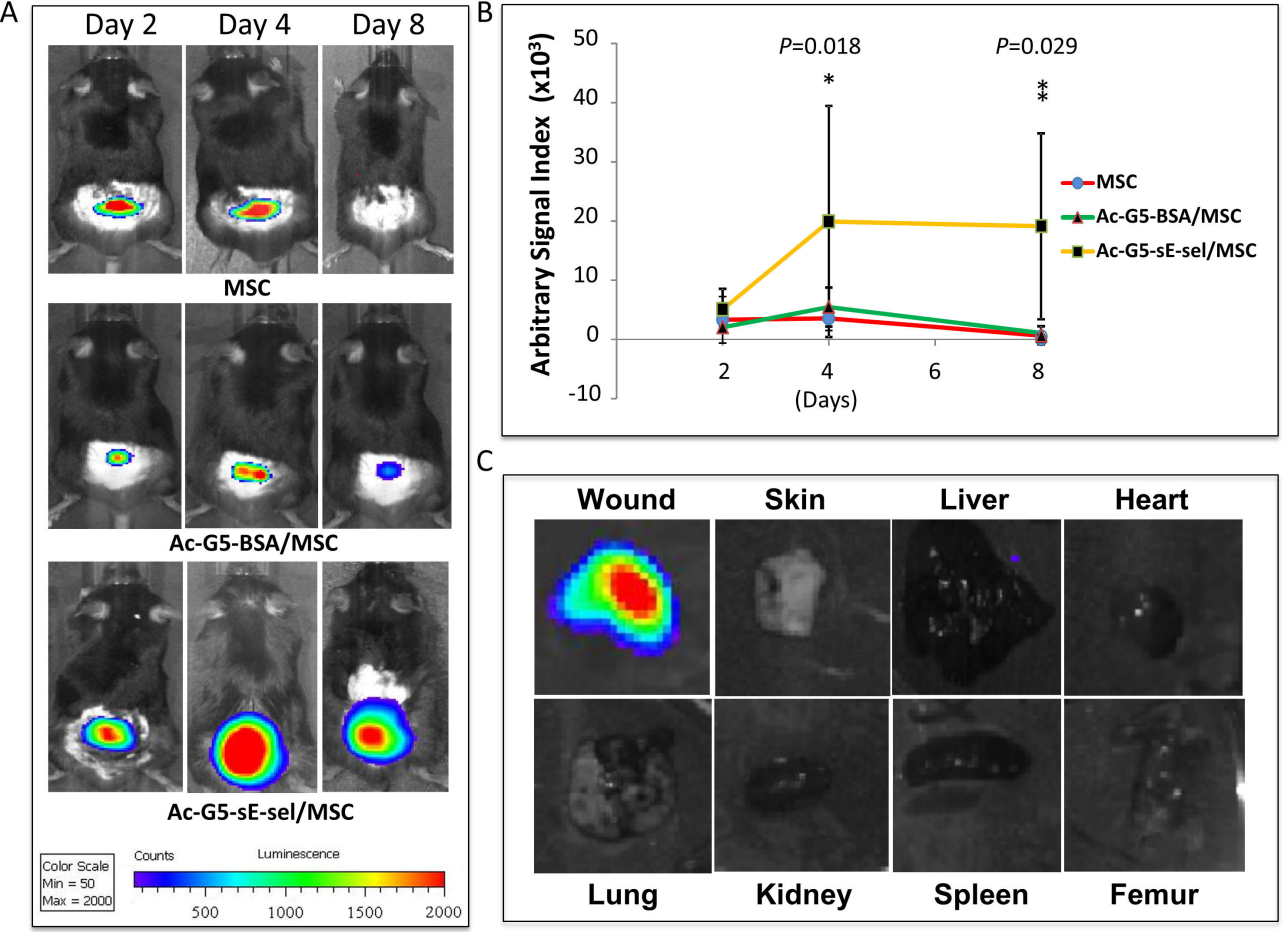
Targeted systemic delivery of nanocarrier-coated MSC to wound tissues. **A**. *In vivo* IVIS imaging shows an increased Ac-G5-dendrimer-sE-sel nanocarrier-coated Luc2^+^-MSC homed to wound tissues compared to either uncoated Luc2^+^-MSC or Ac-G5-BSA-coated Luc2^+^-MSC at various time points. **B**. Quantitative data of bioluminescence signals in each group (n = 4/group) at various time points. **C**. Bioluminescence imaging shows Ac-G5-dendrimer-sE-sel nanocarrier-coated Luc2^+^-MSC selectively homed to skin wound tissues but not other organs, as demonstrated by IVIS scanning of the harvested lung, heart, liver, spleen, kidney, femur, normal and wounded skins on Day 8.

To investigate biodistribution of intravenously infused Ac-G5-sE-sel nanocarriers-coated Luc2^+^-MSC, recipient mice with dorsal skin wounds were subjected to whole-body IVIS scan at day 2, 4 and 8 post i.v. cell infusion to track localization of infused Luc2^+^-MSC. Compared to mice injected with uncoated Luc2^+^-MSC and control BSA nanocarriers-coated Luc2^+^-MSC, mice administered with Ac-G5-sE-sel nanocarriers-coated Luc2^+^-MSC showed significantly increased Luc2^+^-MSC within their cutaneous wound tissues, particularly on day 8. (Fig 6A and 6B). Some uncoated and control nanocarries-coated Luc2^+^-MSC transiently gathered in wound tissues at day 2 and 4, however, only the Ac-G5-sE-sel nanocarriers-coated Luc2^+^-MSC were sustained in wound tissue at day 8. This is consistent with results of IHC for detection of LacZ^+^-MSC described above. Therefore, two different methods (IVIS for Luc2 and IHC for LacZ) demonstrated effectiveness of our Ac-G5-sE-sel nanocarriers-coated cell delivery platform in specifically transporting stem cells to targeted tissues. Most notably, intravenously infused Ac-G5-sE-sel nanocarriers-coated MSC selectively homed to wound tissues, but not other organs as demonstrated by IVIS scanning of the harvested lung, heart, liver, spleen, kidney, femur, normal skin and wounded skin, on day 8 (Fig 6C). Our results suggest that the Ac-G5-sE-sel remains as a complex *in vivo* and these nanocarriers guide MSC to their destination.

## Discussion

**Fig 7:**
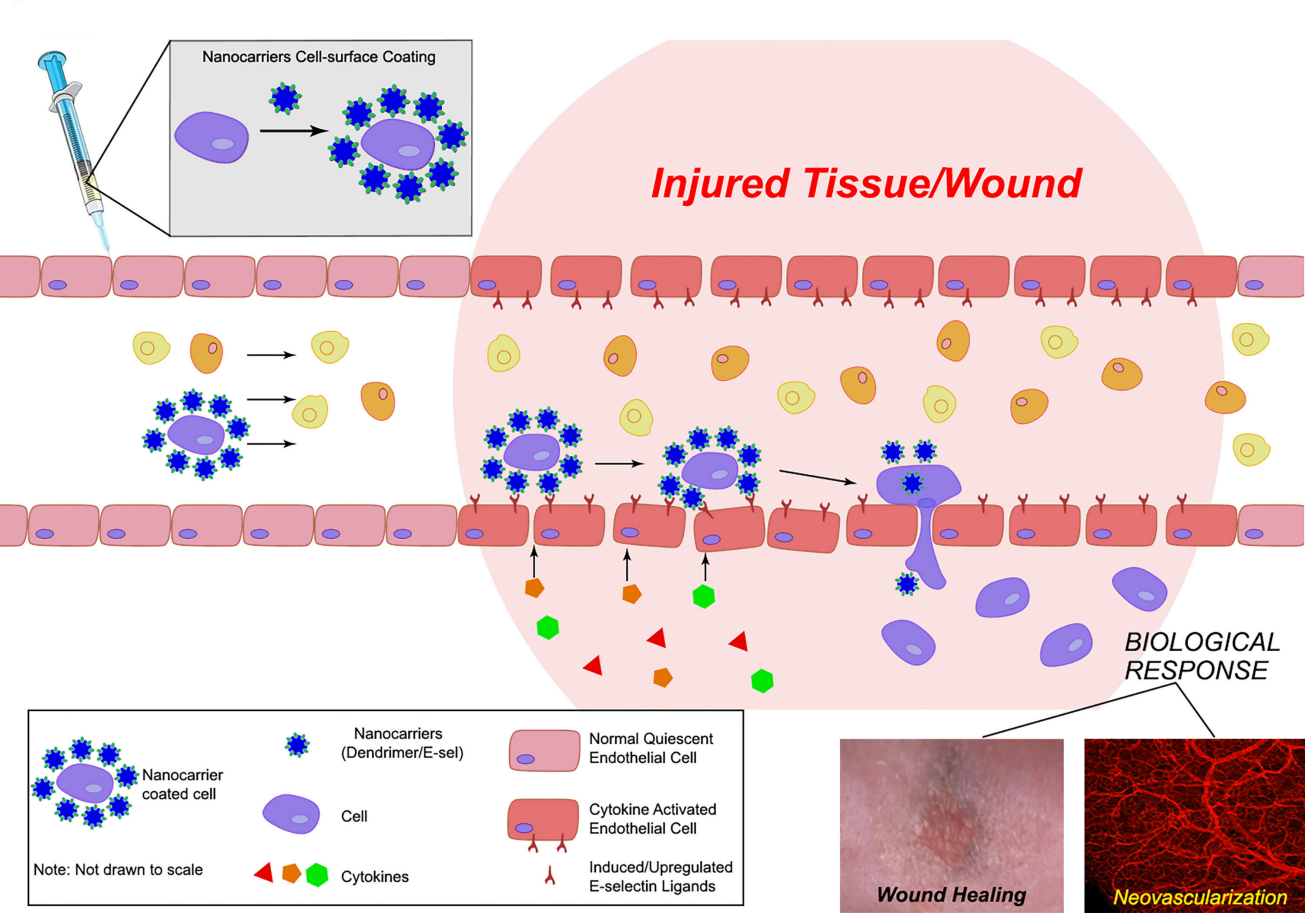
Illustration of this novel targeted systemic cell delivery system. The cell surface is coated with nanocarriers composed of dendrimers conjugated with adhesion molecules. These nanocarriers guide coated cells homing to the desired tissue via association with the counterpart molecules highly or selectively expressed on the endothelium of diseased tissues.

We have developed an innovative nanotechnology-based cell delivery system by coating the cell surface with cell adhesion molecule modified nanocarriers that direct cells to target tissues (Fig 7). We have showed that our nanocarrier-mediated cell delivery is superior to conventional stem cell injections in terms of numbers of cells homed to target tissues and resulting biological response, for example, faster wound healing and enhanced neovascularization. By identification of the highly and/or selectively expressed/activated adhesion molecule(s) on the endothelium in the diseased tissues, and selection of corresponding adhesion moiety to create unique nanocarrier, therapeutic cells can be specifically delivered to the target tissues. Here, we identified E-selectin ligand highly expressed on endothelium in injured tissue and employed sE-sel to create nanocarriers. When selecting adhesion molecule(s)/moieties for conjugation with dendrimers to create nanocarriers, it is important to avoid interaction of ligand/receptor induced pro-inflammatory responses or other unintended signal transduction in luminal EC of the targeted tissues. Thus, theoretically, it may be better to design or use small fragments of adhesion molecule(s), for instance, simple binding moiety, which retains binding capacity yet does not induce a full signaling cascade.

The mechanism by which the nanocarrier interacts with the cell surface is based on ionic interactions. The naocarrier is constituted by a dendrimer that is cationic while the cell surface is anionic. Thus, dendrimer nanocarriers are attached to the cell surface by ionic binding. Since such an association is non-covalent and reversible, it is possible that after cell infusion, dendrimers may dissociate from coated cells and equilibrate with other cells *in vivo*. However, our data showed that systemically administrated nanocarriers-coated cells selectively home to targeted tissues, indicating that dendrimer nanocarries stay on the cells after infusion. This could be attributed to the stabilization of dendrimer nanocarriers on the cell surface by other interactions, such as protein-protein, protein-carbohydrates (nanocarriers interact with other cell surface molecules), or held on the cell surface during the process of cell engulfing (partial internalization of the dendrimer nanocarriers), after initial electrostatic interaction. Eventually, the dendrimer nanocarriers coated on cell membrane can be taken up by cells, discharged/expelled from cells and totally eliminated from the body effectively via renal clearance, thus, having their non-toxic, biocompatible nature [35]. We also demonstrate that our nanocarrier-mediated cell delivery is a safe method and non-toxic, both *in vitro* and *in vivo* (Table A, Figs B and C in S1 File). While it is not known whether the dendrimer nanocarries may have an effect on cellular functions of the coated cells, our study, demonstrates that MSC and BMC coated with Ac-G5-sE-sel nanocarriers retain their tissue repair function (pro-healing and pro-angiogenic).

In theory, the platform can be used to program the targeted delivery of any cell types to any specific tissues, as long as the elements of the adhesion molecule-ligand interactions are designed for the desired clinical translational application. Here, we have investigated and established the effectiveness of these nanocarriers in enhancing three different types of cell-cell interactions using *in vitro* and *in vivo* models, namely, (1) EPC-EC for *in vitro* cell-cell binding; (2) MSC-EC for *in vivo* MSC delivery in a cutaneous wound model, BMC-EC for *in vivo* BMC delivery in a cornea injury model. In the case of BMC, which involves a heterogeneous population of cells, the data shows versatility of the positively charged nanocarriers in coating different lineages of cells by association with their negatively charged cell membrane. Thus, in this case, pure cell populations or full characterization of cells are not required for cell coating. The ability to tailor nanocarriers to carry different types of cells and program the direct delivery to a variety of tissues makes this method versatile and amenable for use in clinically-relevant cell-based therapies in various diseases. Moreover, we envision that this approach may find applications in transplantation or regenerative medicine cell-based treatments. Cells decorated with these nanocarriers containing an imaging agent could also allow for *in vivo* cell tracking.

Our data show that systemically administrated Ac-G5-sE-sel nanocarriers-coated MSC selectively home to wound tissues, but not to other organs. In particular, in cases of intravenously administered cells, the lung and liver are known to create a significant non-specific cell trap because of their large capillary surface area. Our data indicate that interaction between E-selectin ligands, such as CD162 and CD44, which are induced to be highly expressed and activated on the capillaries in wounds, and sE-sel, coated on the cell surface of MSC, is sufficient for mediating capture of the nanocarrier-decorated MSC to the wounded tissues. Many other types of cells, including some circulating blood cells, can express basal levels of CD162 and CD44. However, the observed highly specific tissue homing of coated-MSC suggests that levels and activities of CD162 and CD44 expressed on endothelium in injured tissues are sufficiently high to secure functional association between the coated-MSC and capillaries/postcapillary venules within injured tissues, even under the interruption of dynamic blood flow/shear force of circulation (such as in skin wounds) and in relatively avascular tissue (such as in corneas). In fact, it is likely that relatively avascular and low flow tissues may pose a homing advantage for the nanocarrier-adhesion moiety decorated cells, since in high flow areas, the force of dynamic blood flow may decrease the binding between E-selectin ligand and sE-sel. These shear forces inhibiting specific binding become particularly important, since the designed interactions in this herein reported method are non-covalent and reversible, thus creating a relative advantage for binding within the low flow conditions of injured tissues. It is thus likely that affinity and avidity to secure binding between E-selectin ligand and E-selectin under static status versus dynamic status may be different. Therefore, it is not surprising if basal levels of CD162 or CD44 expressed on the naturally circulating blood cells are insufficient to secure efficient binding with the sE-sel conjugated nanocarriers. Alternatively, it is possible that CD162 or CD44 expressed on certain types of blood cells may not be activated (not glycosylated) so that they are unable to associate with either sE-sel on coated cells or induced endogenous E-selectin on activated endothelium within injured tissues.

In addition to mediate targeted systemic cell delivery, Ac-G5-sE-sel nanocarriers appear to potentiate pro-angiogenic and pro-healing effects of MSC, which are engrafted directly into diabetic wound tissues. The biological responses observed in our experiments could be attributed to the direct effect of pro-repair MSC or BMC systemically delivered or locally injected to the injured tissues, or alternatively, due to the paracrine effects of these delivered cells. The precise mechanism underlying the therapeutic effect has not been elucidated in the current study since its focus is geared toward development of the targeted cell delivery platform. However, future studies will be undertaken to understand the mechanisms for the observed supra-normal cutaneous healing rates achieved with Ac-G5-sE-sel-coated MSC. Normal mice have evolved to heal much faster than humans and thus delayed healing models (by induction of diabetes and/or ischemia) are usually required to demonstrate a biological outcome in wound healing after intervention. To date, no report about accelerated cutaneous wound healing above naturally occurring murine healing cascades. Our findings have identified a new area of fertile investigation that tackles critical unexplored questions, i.e., is it possible that the natural existing regenerative capacity of tissues can be artificially enhanced and, if so, to what extent and by what mechanisms?

## Conclusions

We report a new targeted stem cell delivery method based on cell surface coating with dendrimer-adhesion molecule(s) nanaocarriers. The nanocarriers guide the coated cells reach the targeted tissue location via their programmed molecular recognition, thus acting as a ‘GPS’ in the delivery process. The method allows for specific tissue homing and beneficial biological outcomes with highly relevant translational applicability and utility. The cell coating technology can mediate cell-cell interactions and tissue-targeted cell delivery for therapeutic angiogenesis and wound healing with no evidence of toxicity in *in vivo* murine models, and *in vitro* human cells. The ability to custom design and modify the nanocarrier platform based on specific therapeutic needs endows this approach with versatility and broad potential applicability.

## Supporting Information

S1 File (PDF)
