# Supplementary material for: Directing and Potentiating Stem Cell-Mediated Angiogenesis and Tissue Repair by Cell Surface E-Selectin Coating
Source: PLoS One. 2016 Apr 22;11(4):e0154053. doi: 10.1371/journal.pone.0154053 (PMC4841581; doi:10.1371/journal.pone.0154053)
Supplement: S1 File — (PDF) [file pone.0154053.s001.pdf]

S1 File

Results

Ac-G5-dendrimer-sE-sel nanocarriers potentiate pro-healing effect of MSC *in vivo*.

We examined local delivery of the MSC coated with Ac-G5-dendrimer-sE-sel nanocarrier to a cutaneous excisional wound created in a diabetes mouse (*db/db*) model. 1 x 10^6 allogeneic MSC were coated with Ac-G5-dendrimer-sE-sel nanocarriers or Ac-G5-dendrimer-BSA nanocarriers (control) and directly injected into wound bed. Pro-repair efficacy was assessed by daily wound area measurements via digital photography. A significantly faster wound-healing rate was observed in the wound that received MSC coated with the Ac-G5-dendrimer-sE-sel (Figure A in S1 File). These results demonstrated that cell surface decoration with Ac-G5-dendrimer-sE-sel nanocarrier potentiates pro-healing effect of MSC.

Biosafety of *in vitro* & *in vivo* delivery

We further tested the biosafety of our nanocarrier cell coating method *in vitro* and *in vivo*. First, we investigated the cytotoxicity of the Ac-G5 dendrimer nanocarriers to human cells. The potential toxic effect of the Ac-G5 dendrimer nanocarriers on inducing apoptosis was tested in HUVEC. Flow cytometric detection of early apoptotic HUVEC using Annexin V-FITC and propidium iodide

(PI) confirmed a negligible cell toxicity index for the Ac-G5 dendrimer nanocarrier compared to BSA controls (Figure B-(a) in S1 File).

To investigate the biosafety profile of intravenous injected nanocarrier-coated MSC (Ac-G5-dendrimer-sE-sel-coated murine MSC) in mice, we conducted blood tests and urinalysis. 12-14 week-old C57BL6 mice (n=15) underwent 6 mm full thickness dorsal dermal wounding and then assigned to 3 treatment groups: (i) 1x 10^6 nanocarrier-coated MSC, (ii) 1x 10^6 MSC and (iii) equivalent volume saline (No Treatment (NT)); (n=5/group). At day 8 post-wounding and treatment, comprehensive metabolic panel, complete blood count and urinalysis were obtained. Blood tests and urinalysis revealed that the hematological, hepatic, renal and pancreatic functions were normal in all groups. In Figure B-(b) in S1 File, all tested parameters were within the normal range among three groups and no significant difference, except ALT at day 8 in untreated group was higher than that in Ac-G5-sE-sel-coated MSC and MSC treated groups (P<0.05) and over the range (it is unclear why ALT levels are increased in the untreated group). However, there is no significant difference between Ac-G5-sE-sel-coated MSC and MSC treated groups. In Figure C in S1 File, percentage of lymphocytes in blood of Ac-G5-sE-sel-coated MSC and MSC treated groups is higher than that in untreated group (P<0.05), but stay within the normal range (20-80%). Also, percentage of neutrophils in blood of Ac-G5-sE-sel-coated MSC and MSC treated groups is lower than that in untreated group (P<0.05), but there is no significant difference between Ac-G5-sE-sel-coated MSC and MSC treated groups.

An expected stress-response induced hyperglycemia was observed among all groups at comparable levels (no significance, p=0.136). Hematological and blood/urine parameters were also within normal range in all groups. These results show that intravenous administration of Ac-G5-dendrimer-sE-sel-coated MSC does not appear to cause metabolic or hematologic toxicity in mice.

Materials and Methods

Local injection of MSC in diabetic wound tissues. 1 x 10^6 allogeneic MSC were coated with Ac-G5-dendrimer-sE-sel nanocarriers or Ac-G5-dendrimer-BSA nanocarriers (control) and suspended in 100 µl of PBS. Cells were directly injected into wound bed created in dorsal skin of diabetic mice (*db/db*). Wound healing rate was assessed by daily wound area measurements via digital photography and percent of wound re-epithelialization (covered with new skin) was calculated using Image J.

Apoptosis Assay. Difference in cell apoptosis between MSC coated with Ac-G5-sE-sel or Ac-G5-BSA versus BSA was determined using flow cytometry. After 2 hours incubation with either Ac-G5-BSA or BSA, apoptosis was determined using a FITC-conjugated annexin-V propidium iodide kit (Abcam) according to the manufacturer's instructions.

Blood tests and urinalysis toxicity studies. Mouse blood were collected at day 8 post post-treatment and tested by core facility of University of Miami using standard methods.

Figure Legends

Table A. The cytotoxicity of Ac-G5-PAMAM and various dendrimers and modification conditions toward human umbilical vein endothelial cells (HUVEC). Trypan blue exclusion cell viability assays demonstrated that Ac-G5 dendrimer nanocarriers did not cause HUVEC cytotoxicity. Different formulations of dendrimers were incubated with human endothelial cells, after 30 min at Room Temperature, the percentage of dead cells were counted by trypan blue staining. The cargo: Ac-G5 at ratio 5:1 did not result in cell toxicity.

Figure A. Pro-healing effect of direct wound tissue injection of Ac-G5-sE-sel nanocarriers-coated MSC. Healing rate expressed as percent of wound re-epithelialization (covered with new skin). *Top*: representative wounds at different days are shown for each group. *Bottom*: wound healing rate. Data are percentage of mean ± SD (n=6 mice/group).

Figure B. *In vitro* and *in vivo toxicity* assays of Ac-G5-dendrimer nanocarrier. (**a)** shows flow cytometric detection of early apoptotic HUVEC treated with Ac-G5-BSA nanocarrier and BSA, respectively, confirming a negligible cell toxicity index for the Ac-G5 dendrimer nanocarrier comparable to that of BSA control. (**b)** shows the metabolic profile of blood test in mice intravenously infused with MSC

coated with Ac-G5-sE-sel nanocarrier or controls. * indicates the difference between two groups is statistically significant (P<0.05).

Figure C. Blood of mice treated with Ac-G5-sE-sel-coated MSC were obtained on day 8 post treatment and were subjected to test for a variety of hematological and blood chemistry and parameters, which reflect the function of kidney, pancreas and liver. These data indicate that Ac-G5-sE-sel-coated MSC have acceptable or no toxicity to mice and are safe. * indicates the difference between two groups is statistically significant (P<0.05).

Table A. The cytotoxicity of Ac-G5-PAMAM and various dendrimers and modification conditions toward human umbilical vein endothelial cells (HUVEC).

Condition	*% of Dead Cells*
G5 1:1	14
G5 1:5	12
G5 5:1	23
G3 1:1	11
G3 1:5	13
G3 5:1	22
Ac-G5 1:1	27
Ac-G5 1:5	5
Ac-G5 5:1	34
G5 alone	32
G3 alone	11
Ac-G5 alone	29
BSA alone (Base line)	13

Figure A

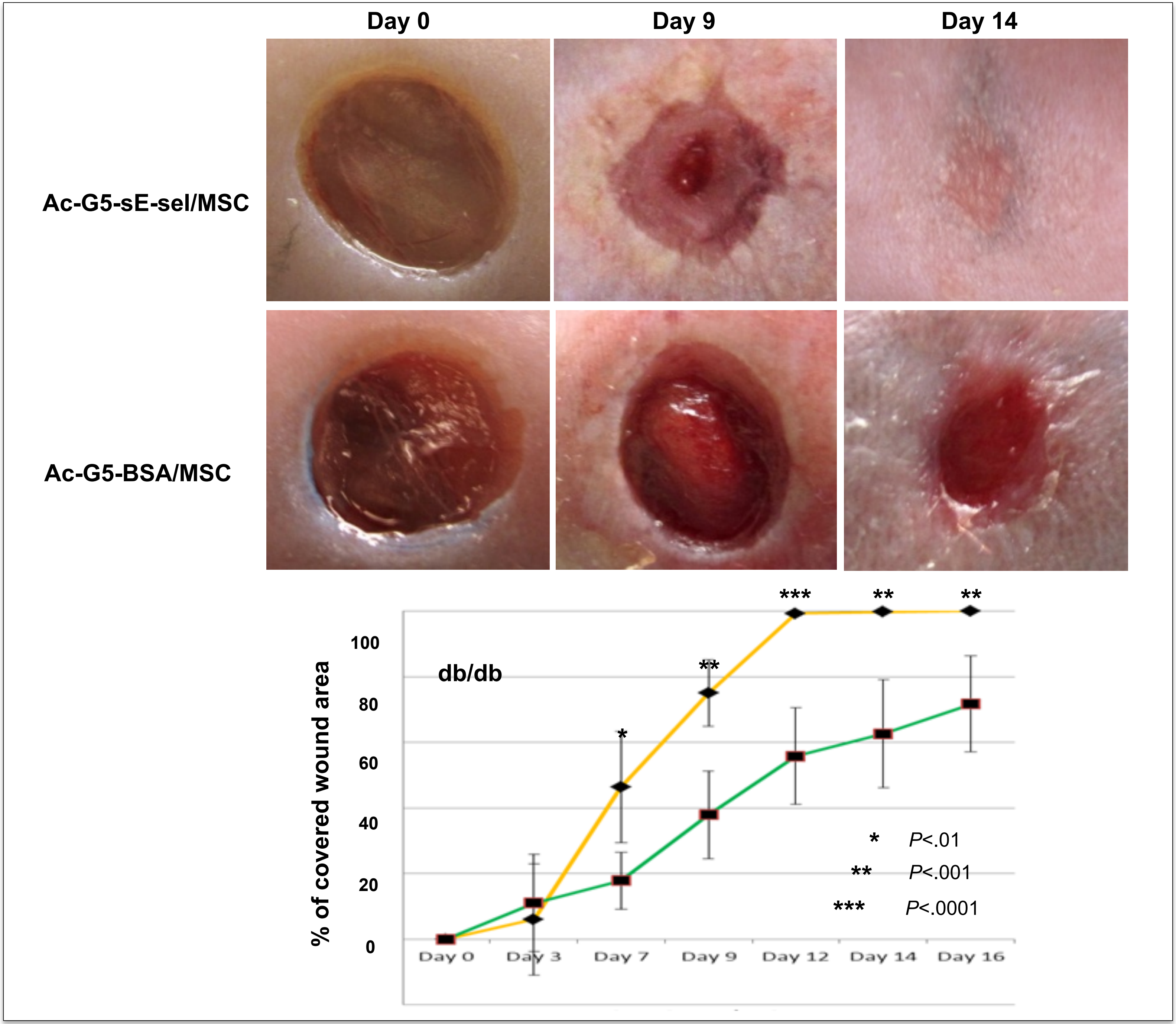

Day 0
Day 9
Day 14
Ac-G5-sE-sel/MSC
Ac-G5-BSA/MSC
% of covered wound area
db/db
100
80
60
40
20
0
Day 0
Day 3
Day 7
Day 9
Day 12
Day 14
Day 16
*
**
***
**
**
* P<.01
** P<.001
*** P<.0001

Figure B

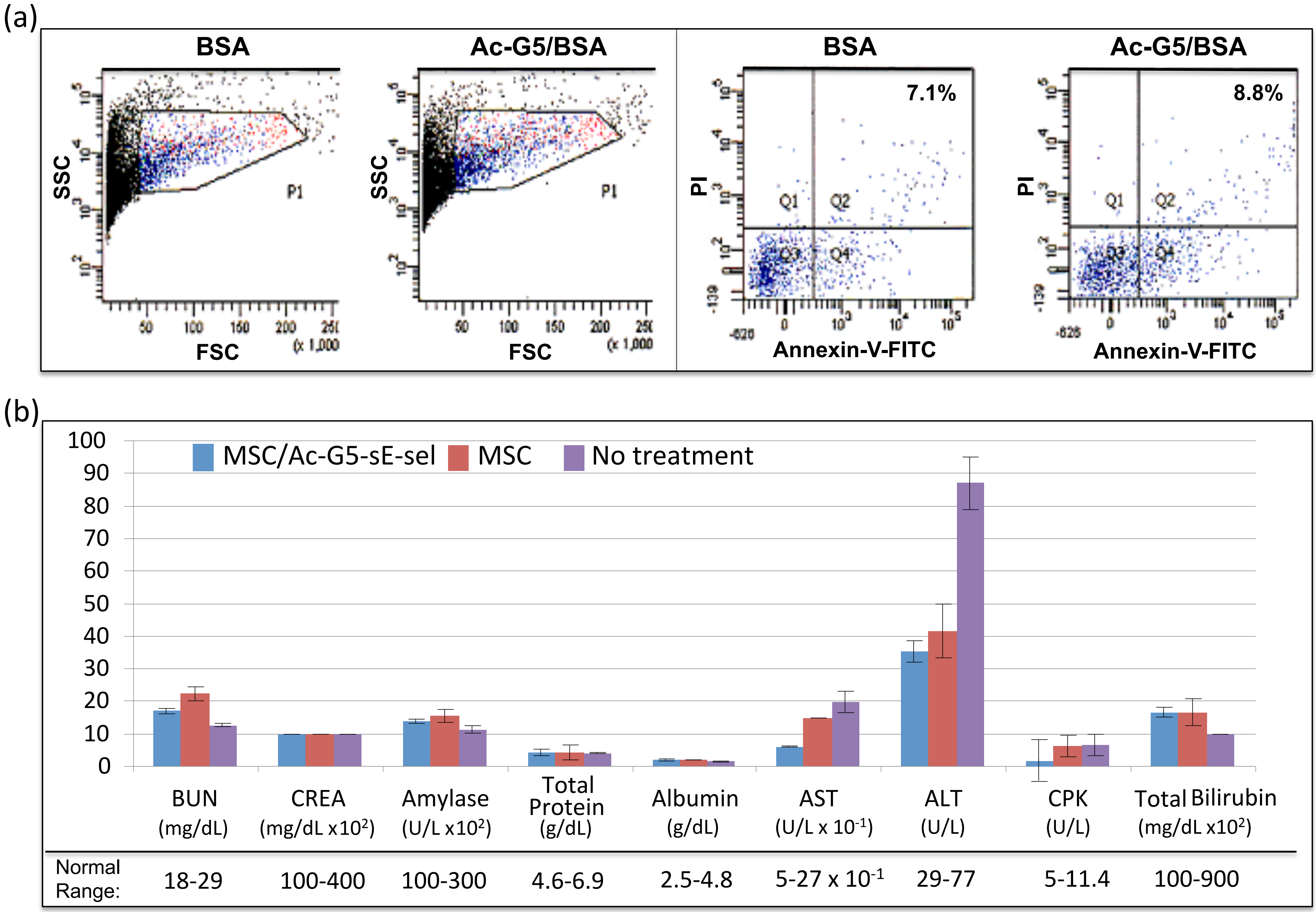
(a)
BSA
Ac-G5/BSA
SSC
FSC
P1
(x 1,000)
BSA
Ac-G5/BSA
7.1%
8.8%
PI
Q1
Q2
Q3
Q4
Annexin-V-FITC
(b)
MSC/Ac-G5-sE-sel
MSC
No treatment
100
90
80
70
60
50
40
30
20
10
0
BUN (mg/dL)
CREA (mg/dL x10^2)
Amylase (U/L x10^2)
Total Protein (g/dL)
Albumin (g/dL)
AST (U/L x 10^{-1})
ALT (U/L)
CPK (U/L)
Total Bilirubin (mg/dL x10^2)
Normal Range:
18-29
100-400
100-300
4.6-6.9
2.5-4.8
5-27 x 10^{-1}
29-77
5-11.4
100-900

Figure C

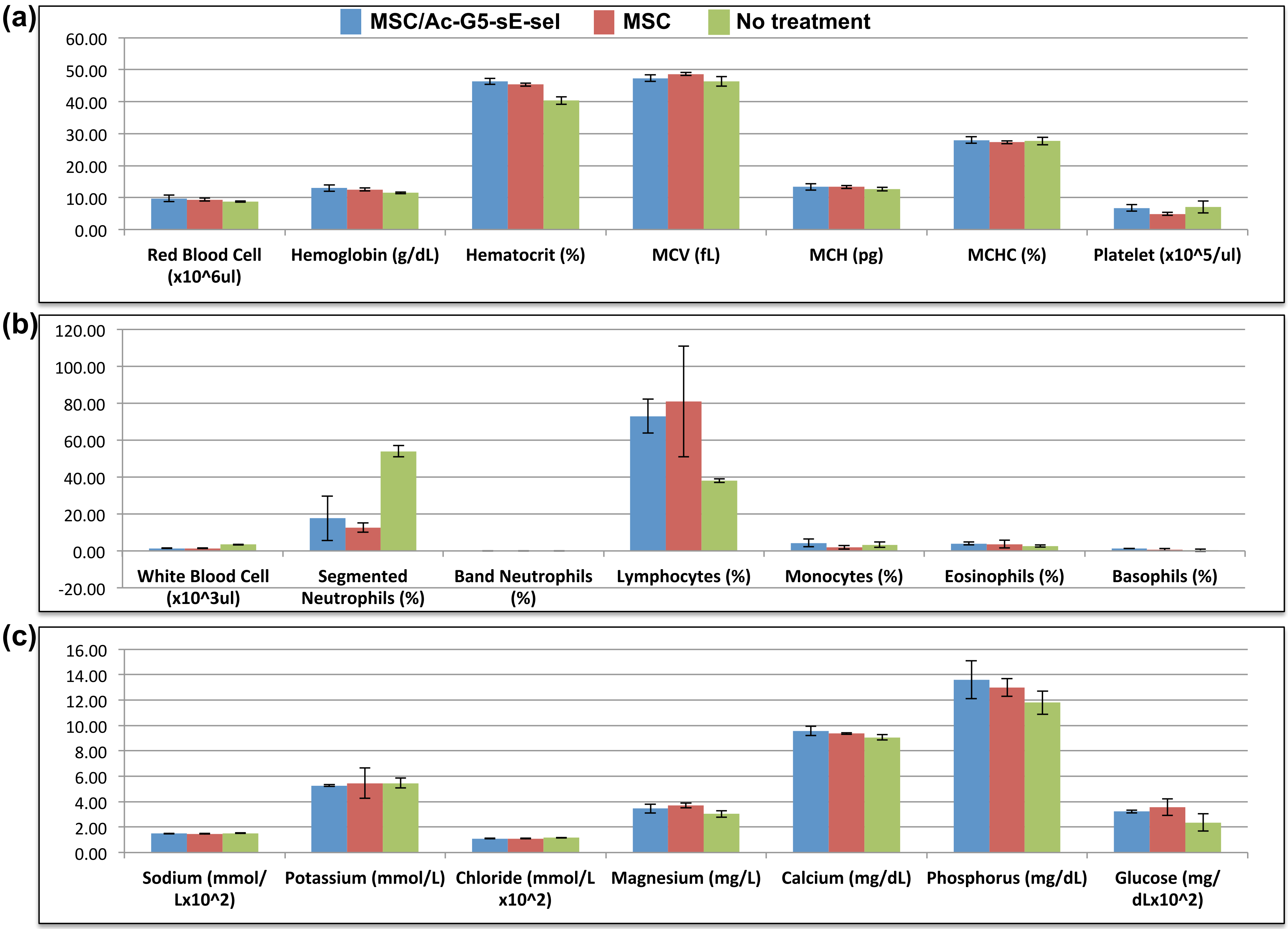

(a)
MSC/Ac-G5-sE-sel
MSC
No treatment
60.00
50.00
40.00
30.00
20.00
10.00
0.00
Red Blood Cell (x10^6ul)
Hemoglobin (g/dL)
Hematocrit (%)
MCV (fL)
MCH (pg)
MCHC (%)
Platelet (x10^5/ul)
(b)
120.00
100.00
80.00
60.00
40.00
20.00
0.00
-20.00
White Blood Cell (x10^3ul)
Segmented Neutrophils (%)
Band Neutrophils (%)
Lymphocytes (%)
Monocytes (%)
Eosinophils (%)
Basophils (%)
(c)
16.00
14.00
12.00
10.00
8.00
6.00
4.00
2.00
0.00
Sodium (mmol/ Lx10^2)
Potassium (mmol/L)
Chloride (mmol/L x10^2)
Magnesium (mg/L)
Calcium (mg/dL)
Phosphorus (mg/dL)
Glucose (mg/ dLx10^2)
